# Biological Rationale for the Use of PPARγ Agonists in Glioblastoma

**DOI:** 10.3389/fonc.2014.00052

**Published:** 2014-03-14

**Authors:** Hayley Patricia Ellis, Kathreena Mary Kurian

**Affiliations:** ^1^School of Cellular and Molecular Medicine, University of Bristol, Bristol, UK; ^2^Brain Tumour Research Group, Institute of Clinical Neuroscience, University of Bristol, Bristol, UK

**Keywords:** glioblastoma multiforme, PPAR gamma, brain tumour, thiazolidinediones, glioma

## Abstract

Glioblastoma multiforme (GBM) is the most common primary intrinsic central nervous system tumor and has an extremely poor overall survival with only 10% patients being alive after 5 years. There has been interesting preliminary evidence suggesting that diabetic patients receiving peroxisome proliferator-activated receptor gamma (PPARγ) agonists, a group of anti-diabetic, thiazolidinedione drugs, have an increased median survival for glioblastoma. Although thiazolidinediones are effective oral medications for type 2 diabetes, certain agonists carry the risk for congestive heart failure, myocardial infarction, cardiovascular disease, bone loss, weight gain, and fluid retention as side-effects. The nuclear receptor transcription factor PPARγ has been found to be expressed in high grade gliomas, and its activation has been shown to have several antineoplastic effects on human and rat glioma cell lines, and in some instances an additional protective increase in antioxidant enzymes has been observed in normal astrocytes. At present, no clinical trials are underway with regards to treating glioma patients using PPARγ agonists. This review presents the case for evaluating the potential of PPARγ agonists as novel adjuvants in the treatment of refractory high grade glioma.

## Introduction

The PPARs are ligand-inducible transcription factors of the nuclear receptor superfamily (1). There are three PPARs expressed in mammalian tissues: PPARα is primarily expressed in the heart, liver, and brown adipose tissue; PPARβ/δ is ubiquitously expressed; and PPARγ is most highly expressed in white and brown adipose tissue (2).

The PPARs control complex gene expression involved in lipid metabolism and adipogenesis, as well as inflammation, and metabolic homeostasis (3). Under healthy conditions, PPARs are primarily receptors for dietary fats such as oleic, linoleic, and linolenic acids, and also bind diverse lipid metabolites, for example prostaglandin J2, 8S-hydroxyeicosatetraenoic acid, and oxidized phospholipids (4). Ligand binding induces a conformational change in the receptor that allows modulation of PPAR activity via differential recruitment of cofactors and histone modification enzymes. For a detailed account of PPARγ signaling and metabolism, see the review by Ahmadian (5).

Recent studies using high throughput genome-wide transcriptional regulation techniques have now revealed the comprehensive distribution of binding-sites of PPARγ in adipocytes and macrophages (6). PPARγ has been found to be key for adipocyte differentiation using PPARγ knockout mice, which are entirely devoid of adipose tissue (7). All PPARs have been isolated from both developing and adult brain tissue (8), and it has been postulated that activation of the PPAR pathway could have a role in determining neuron viability in the developing midbrain (9).

**Figure 1:**
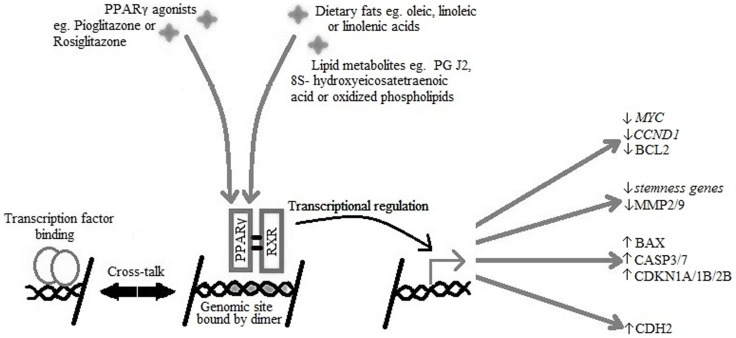
**PPARγ and RXR heterodimer binding to the PPARγ genomic binding site in DNA causing transcriptional activation of target genes active in cell-cycle arrest, reduced expression of stemness markers, initiation of apoptosis, and re-differentiation**.

PPARγ has two isoforms due to differential promoter usage and alternative splicing: PPARγ1 which is expressed in many tissues and PPARγ2, which under normal conditions is present in adipose tissue, but can be induced by a high fat diet to be expressed in other tissues. PPARγ forms a heterodimer with retinoid-X-receptor (RXR) to bind ligand efficiently, after which the receptor ligand complex binds DNA and induces signal transactivation (10) (see Figure 1).

## PPARγ in Neoplasia

A recent retrospective clinical review by Grommes showed that diabetic GBM patients treated with PPARγ agonists exhibited an increased median survival of 19 months compared to patients receiving the standard treatment for GBM alone, for whom median survival was 6 months (11). However, the group of patients eligible for statistical analysis was small so the negative correlation between PPARγ agonist use and GBM development was not found to be significant (11). A study has been conducted to determine whether there is a somatic mutation of the *PPARG* gene with high penetrance that might play a role in the development of GBM (12). Although no high penetrance mutations were found, two polymorphisms were identified, one at codon 12 (*PPARG^P12A^*) and the other at codon 449 (*PPARG^H449H^*) of the *PPARG* gene, and these were found to be highly over-represented in patients with GBM when compared to the matched control group (12). Thirty-three percent of the GBM patients were found to be heterozygous for the *PPARG^P12A^* allele (a CCA to GCA polymorphism causing a change from proline to alanine) (12). The *PPARG^H449H^* polymorphism (a CAC to CAT change, though the amino acid remains a histidine) appeared to have much higher levels of over-representation, as it demonstrated homozygous expression in 50% of GBM patients and only 12% of the control group (12).

The study was repeated with German participants and no deviation was found from the normal representation in the population of either polymorphism (12). However, this could be explained by the variants in the original study being in a linkage disequilibrium, which would be corollary to the founder effect with a relatively new founding allele (12). Preliminary analysis of the *PPARG^P12A^* and *PPARG^H449H^* polymorphisms found that there was no over-representation or under-representation of these alleles in patient populations with other types of cancers (e.g., melanoma or breast), which suggests that the effect is specific for glioma (12).

PPARγ expression has been described in a range of other neoplasias including colon, lung, prostate, bladder, breast, duodenal, and thyroid (13–19). Interestingly in colon cancer there are differing older reports as to the effect of PPARγ agonists. Some *in vitro* studies describe differentiation, reduction of malignancy, and inhibition of anchorage-independence in colon cancer (20) whereas in other mouse models enhancement of polyp formation has been observed (21). It has been put forward by Sarraf et al. (13) that PPARγ exhibits tumor suppressive activities in colon cancer because several functionally deleterious *PPARG* mutations have been found in cases of sporadic colon cancer. However, in the case of colon cancers with known deletions in the *APC* (adenomatous polyposis coli) tumor suppressor gene, PPARγ agonists appear to promote tumor growth, and increase the number of colon polyps, possibly by increasing the uptake of dietary fat (21). In human bladder cancer, PPARγ agonists troglitazone and 15d-PGJ2 have shown to inhibit tumor growth (22). By contrast, in an investigation in rats of the effect of Naveglitazar, a PPARα/γ dual agonist showed a significant increase in bladder neoplasms. (23). In another study to determine if rosiglitazone had chemopreventive activity, female rats were treated with different doses of rosiglitazone plus a urinary bladder-specific carcinogen, and it was found that larger cancers developed compared with rats treated with the bladder carcinogen alone (24). However, no apparent activity of rosiglitazone as a complete carcinogen was observed. (24). Additionally, the effects were only recorded in females and it was postulated that this could be due to irritant effects. However, the effects were observed rapidly after administration with rosiglitazone, contesting a long-term chemical irritant effect (24).

It is important to mention that many of the carcinogenic effects of the agonists for the nuclear receptor PPARγ are highly species specific; i.e., observed in rodents but not humans or other higher order mammals.

## Current Research into PPARγ Agonists and Glioblastoma Multiforme

### Brain tumor growth inhibition

One of the important hallmarks of cancer is a proliferative advantage over normal tissue. One possible mechanism by which PPARγ agonists can inhibit cell proliferation is by induction of cell-cycle arrest in G0/G1 phase (25–27), and a reduction of the proportion of cells entering S-phase (25, 27). In concordance with this finding, decreased levels of MYC have also been detected upstream of the S-phase transition (25, 28), as well as possible down-regulation of CCND1 (cyclin D1) and associated cyclin-dependent kinases (25, 28). The decreased proportion of cells entering S-phase in response to PPARγ agonists has also been linked to up-regulation of the cyclin-dependent kinase inhibitors CDKN1A, CDKN1B, and CDKN2B (25, 27).

PPARγ agonists have also been found to inhibit the expansion and proliferation of CD133^+^ brain tumors stem cells (BTSCs, also termed Brain Tumor Initiating Cells) by inhibiting the Janus kinase/signal transducer and activator of transcription (JAK/STAT) pathway using ciglitazone, 15-deoxy-Δ12,14-prostaglandin J2 (15d-PGJ2), and all-trans retinoic acid (ATRA) (26, 29).

JAK/STAT signaling is particularly important in the anti-tumor activity of PPARγ agonists because the inhibition of JAK2 (upstream regulator of STAT3) has been shown to have a role in slowing the disease progression of GBM *in vivo* and *in vitro* models (30), and troglitazone has been described as an antagonist for STAT3 signaling (31). Antagonists for the JAK/STAT pathway work by phosphorylating tyrosine 705 of STAT3 leading to down-regulation of CCND1 and BCL2L1 (B-cell lymphoma protein 2 extra large) which act to push cells through the cell-cycle and prevent apoptotic cell death (30). High levels of STAT3 signaling have been noted in BTSCs, and inhibition of this has been shown to decrease BTSC resistance to temozolomide (32), which is promising with regards to use of PPARγ agonists as an adjuvant therapy in GBM. Glioma cell lines have been found to have a much higher rate of cellular metabolism, which can be significantly increased by addition of troglitazone (33). Therefore, glioma cells will suffer nutrient deprivation and could be more susceptible to cytotoxic killing than normal astrocytes (33), an effect which is thought to be mediated by reactive oxygen species produced by mitochondria (34).

### Glioma cell differentiation

One of the ways that PPARγ could counteract the malignant properties of BTSCs is via regulation of genes involved in maintaining a stem cell state termed “stemness genes” as well as differentiation. This is important in the cases of grade III and IV glioma, as the BTSCs may be responsible for the recurrence and growth of the malignant tissue through mechanisms such as self-renewal (35, 36).

Many stemness genes have been found to be down-regulated by activation of the PPARγ pathway by the agonists ciglitazone and 15-PGJ2, though ATRA treated cells showed no significant difference. An example of a stemness gene down-regulated by PPARγ agonists is *SOX2*, which is important in maintaining pluripotency in stem cells, and plays a role in repressing neural differentiation as it is over-expressed in BTSCs but remains at low levels of expression in normal tissue (37). In studies assessing the decrease in expression of stemness genes, mouse neural stem cells were cultured using epidermal growth factor and basic fibroblast growth factor to stimulate expression of stemness markers, and the changes in levels of expression were analyzed using TaqMan low density gene arrays following treatment with PPAR agonists (35, 36). Most stemness genes monitored exhibited down-regulated expression; however expression of *NOG* appeared to be up-regulated, possibly because it is involved in neurogenesis and developmental patterning of the brain across the anterior–posterior axis (38). It has been suggested that the change in expression of stemness genes means that PPARγ agonists can modulate differentiation via regulation of stemness factors (35, 36).

Another way that PPARγ has been found to regulate differentiation of neural stem cells is by inducing expression of differentiation genes (35, 36). Expression of differentiation markers was also analyzed in similar experiments to those described above, and it was found that PPARγ agonists increased expression of glial cell markers in T98G and DB29 BTSCs. Compared to a dimethyl sulfoxide control, ciglitazone, ATRA, and 15d-PGJ2 all resulted in an increased expression of genes such as *GFAP* (glial fibrillary acidic protein) and *TUBB3* (βIII-tubulin), specific to neuronal cells (35, 36). Also, human and rat cell lines treated with PPARγ agonists have been found to transiently increase their expression of CDH2 (N-cadherin), a neural differentiation marker, as well as showing outgrowth with a morphology similar to that of normal astrocytes (39). This suggests that activation of the PPARγ pathway can control differentiation of neural progenitor cells via modulating expression of neural differentiation genes as well as those involved in maintaining pluripotency (35, 36).

### Reduction of local invasiveness

Another effect of PPARγ agonists on glioma tissue is to reduce local tissue invasiveness (28, 40, 41). Invasion of malignant cells into nearby healthy brain tissue in glioma patients may be mainly mediated by matrix metalloproteinases MMP-2 and MMP-9 which exhibit elevated expression in tumor progression (42). MMP-2 and MMP-9 expression has been monitored before and after administration of pioglitazone using immunohistochemical assays (40, 41). Both *MMP-2* and *MMP-9* have independently been found to be down-regulated after treatment with pioglitazone, suggesting the role of PPARγ agonists in reducing glioma cell invasiveness (28, 40, 41). Also, as differentiation marker expression increases in expression after addition of PPARγ agonists, this implies that the cellular phenotype becomes less invasive via differentiation as well as reduction of expression of malignant cell markers (41).

Pioglitazone has also been found to reduce CTNNB1 (β-catenin) expression without changing its cellular localization (28). CTNNB1 controls the expression levels of CDH1 (E-cadherin) to mediate cellular attachments and is often over-expressed in high grade glioma when compared to low grade or normal tissue (43). This is a possible mechanism by which reduction of CTNNB1 expression by PPARγ agonists could contribute to inhibition of the loss of cellular attachments, as well as reducing the transcription of tumor-promoting target genes of CTNNB1 such as *CCND1* and *MYC* (28).

### Induction of apoptosis

One of the most well understood responses of glioma cells to PPARγ agonists is a reduction of cellular viability which leads to the induction of apoptosis (26–28, 33, 39, 41). Many papers have shown that treatment of cell lines with pioglitazone and related TZDs can lead to specific apoptosis of glioma cells in a concentration-dependent fashion associated with cell-cycle arrest, while sparing normal primary astrocytes (26–28, 33, 39, 41).

This effect could be mediated by BAX-dependent mechanisms, as BAX up-regulation is often detected via an increase in protein levels after activation of PPARγ (25, 27, 39, 41). Furthermore, studies have shown that transfection of cells with antisense oligonucleotides for *BAX* nullified the effect of the PPARγ agonist treatment (39). Alternatively, BCL2 has been found to be down-regulated following administration of the same treatment (25, 27). Increases of protein activity of major executioner caspases CASP3 (27, 33, 41) and CASP7 (33) have also been described in response to a PPARα/γ dual agonist and troglitazone.

### Catalase activity

Catalase is an enzyme involved in the neutralization of reactive oxygen species and its gene contains a PPAR genomic binding site (44). Studies have suggested that the cytotoxic effect of PPARγ agonists on glioma cells is partially mediated by enhanced redox reactions (44).

In *in vitro* rat cell models, activation of PPARγ transcription has been shown to upregulate catalase activity in normal astrocytes, but not in the glioma C6 cell line (44).Moreover, this effect was abolished in cells transfected with a dominant negative PPARγ construct (44).

This area requires further experimentation using human cell lines, as catalase could be a possible redox-dependent target for protection of normal astrocytes from the reactive oxygen species produced by intensive therapies such as radiation.

## Limiting Factors

**Figure 2:**
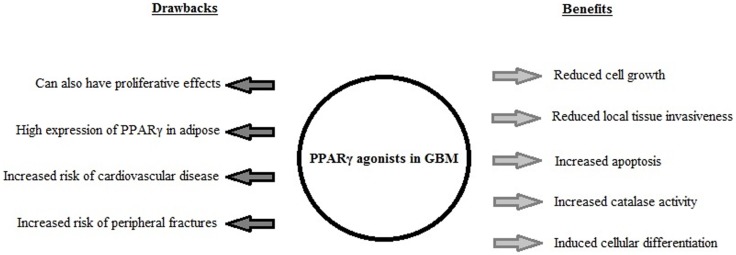
**A summary of the biological effects induced by PPARγ agonist use in GBM *in vitro* and *in vivo*, and the drawbacks associated with PPARγ agonist use in clinical treatment of diabetes mellitus**.

Rosiglitazone and pioglitazone are currently the only TZDs FDA-approved for clinical use, summarized in Table 1. It has been found that use of certain thiazolidinediones in treatment of diabetes mellitus can increase risk of congestive heart failure, myocardial infarction, cardiovascular disease, bone loss, weight gain, and fluid retention. Meta-analysis of eight studies involving 945,286 patients found that compared to pioglitazone, rosiglitazone administration conferred an increased risk of overall mortality as well as heart failure and myocardial infarction (4). The findings of this meta-analysis and the issue of cardiovascular side-effects due to administration of rosiglitazone remains controversial, and this has lead to a dramatic reduction in its use in clinical treatment of diabetes (45) with some countries withdrawing it from the market altogether. Additionally the doses used experimentally to activate the antineoplastic effects mediated by PPARγ have been observed to be much higher than the doses used in treatment of diabetes, even with differences of orders of magnitude (19, 35). The constitutively high levels of PPARγ expression in adipose tissues (when compared to expression levels in the brain) raises the possibility of systemic adverse side-effects from off-target activation of the PPARγ pathway by TZDs in increased. The biological benefits and detrimental side-effects of PPARγ use in GBM are summarized in Figure 2.

**Table 1:** **A summary of the PPARγ agonists referred to in this review and their current status with regards to clinical application**.

PPARγ agonist	Clinical use	Extra information
Pioglitazone	FDA-approved for diabetes mellitus type II	Activates PPARγ to increase insulin sensitivity, also activates PPARα to alter lipid metabolism (46)
Rosiglitazone	FDA-approved for diabetes mellitus type II	Reduced use due to increased association with myocardial infarction and death compared to pioglitazone (47)
Troglitazone	Withdrawn due to severe liver complications	Had additional anti-inflammatory effect as well as antioxidant effects via PPARα and PPARγ activation (48)
Ciglitazone	N/A	The prototypic glitazone in treatment of diabetes from which other PPAR agonists were designed (49)
15d-PGJ2	N/A	Prostaglandin recognized as the endogenous ligand for the PPARγ receptor (50)

In conclusion, we examined the biological rationale for the use of PPAR agonists in glioblastoma, in particular brain tumor growth inhibition, glioma cell differentiation, inhibition of apoptosis, and increased catalase activity. The potential for the use of these agents in this GBM may be limited by recently described side-effects in this group of agents, and the variation between expression levels of PPARγ in different tissues.

However, in patients with such a universal poor prognosis further investigation into this pathway is justified on the basis of preliminary epidemiological data. Advances in knowledge of the PPAR pathway in GBM may identify new cellular targets for brain tumor therapies.

## Conflict of Interest Statement

The authors declare that the research was conducted in the absence of any commercial or financial relationships that could be construed as a potential conflict of interest.
